# Gene Screening for Prognosis of Non-Muscle-Invasive Bladder Carcinoma under Competing Risks Endpoints

**DOI:** 10.3390/cancers15020379

**Published:** 2023-01-06

**Authors:** Chenlu Ke, Dipankar Bandyopadhyay, Devanand Sarkar

**Affiliations:** 1 Department of Statistical Sciences and Operations Research, Virginia Commonwealth University, Richmond, VA 23284, USA; 2 Department of Biostatistics, Virginia Commonwealth University, Richmond, VA 23219, USA; 3 Department of Human Genetics, Virginia Commonwealth University, Richmond, VA 23219, USA

**Keywords:** bladder cancer, competing risk endpoints, gene screening, subdistribution hazards, survival analysis

## Abstract

**Simple Summary:**

A vital task in contemporary cancer research is to discover clinically useful molecular markers for diagnosis and prognosis from microarray or sequencing data. However, reliable and efficient statistical tools are lacking in terms of marker screening and selection for high-throughput data with complicated survival endpoints, such as competing risks. Motivated by a study on progression of non-muscle invasive bladder carcinoma for 300 subjects with competing risk endpoints, this paper proposed a controlled screening procedure to fast eliminate most of irrelevant markers, before more precise selection can be further pursued. Combining screening with a boosting procedure, a significant six-gene signature for progression was identified subsequently, showing improved prediction performance over existing alternatives at a lower computational cost. The proposed method is readily applicable to other types of high-throughput cancer data with competing risk events, providing a desired addition to a biomedical researcher’s toolbox.

**Abstract:**

Background: Discovering clinically useful molecular markers for predicting the survival of patients diagnosed with non–muscle-invasive bladder cancer can provide insights into cancer dynamics and improve treatment outcomes. However, the presence of competing risks (CR) endpoints complicates the estimation and inferential framework. There is also a lack of statistical analysis tools and software for coping with the high-throughput nature of these data, in terms of marker screening and selection. Aims: To propose a gene screening procedure for proportional subdistribution hazards regression under a CR framework, and illustrate its application in using molecular profiling to predict survival for non-muscle invasive bladder carcinoma. Methods: Tumors from 300 patients diagnosed with bladder cancer were analyzed for genomic abnormalities while controlling for clinically important covariates. Genes with expression patterns that were associated with survival were identified through a screening procedure based on proportional subdistribution hazards regression. A molecular predictor of risk was constructed and examined for prediction accuracy. Results: A six-gene signature was found to be a significant predictor associated with survival of non–muscle-invasive bladder cancer, subject to competing risks after adjusting for age, gender, reevaluated WHO grade, stage and BCG/MMC treatment (*p*-value < 0.001). Conclusion: The proposed gene screening procedure can be used to discover molecular determinants of survival for non–muscle-invasive bladder cancer and in general facilitate high-throughput competing risks data analysis with easy implementation.

## 1. Introduction

Bladder cancer is a common type of cancer associated with high morbidity and mortality [1] rates, if not treated optimally. The disease presents in two different forms: non–muscle-invasive (NMI) tumors (stages Tis, Ta and T1), where the cancer is in its early stages, with cells only appearing in the inner lining of the bladder (and have not grown into the deeper bladder muscle layers), and muscle-invasive cancers (stages T2–T4), where cancer cells have spread into the detrusor muscle of the bladder. A variety of treatment protocols exist, stratified on the degree of risk of the disease, such as complete resection of the tumor followed by induction, and maintenance immunotherapy through intravesical BCG vaccine, chemotherapy, transurethral resection, and cystectomy. Most bladder cancers are diagnosed at an early stage, when the cancer is highly treatable. The non–muscle-invasive tumors account for roughly 75% of newly diagnosed cases and 50% of non-muscle-invasive bladder cancers (NMIBC) are low grade [2]. However, even early-stage bladder cancers can recur after successful treatment; more than 60% of patients experience recurrence, and some patients develop muscle-invasive tumors over time [3]. Therefore, early diagnosis with personalized treatment and follow-up is the key to a successful outcome.

Developments in microarray and sequencing technologies have allowed the collection of massive genomic information that substantially advances the understanding of molecular mechanisms, biomarker discovery, and personalized medicine. High-throughput data produced by those techniques are characterized by a large number of features that far exceeds the sample size (p>>n). In clinical studies, a vital research task is to find predictive features for survival outcomes and build prognostic models for cancer patients, which often requires techniques that were developed for specific time-to-event responses. In bladder cancer, one primary endpoint is time-to-progression, but competing events such as death from non-cancer causes can also be observed [4]. The proportional subdistribution hazard (PSH) model proposed by Fine and Gray [5] has become a popular semi-parametric model for competing risks (CR) data. On the basis of the PSH model, feature selection approaches have been developed for high dimensional data (p>n) including LASSO-type methods [6,7,8,9,10] and component-wise boosting [11], among others. These techniques have been used in identifying gene signatures for predicting bladder cancer progression [6,12]. However, when it comes to ultrahigh dimensional data (p>>n), exact feature selection is presumably very challenging, if not impossible, to achieve. The aforementioned methods may become statistical inaccurate and computationally expensive [13].

Recent years have seen an increasing attention to feature screening as a preliminary procedure to fast filter out unimportant features before using penalized or boosting methods for more precise selection. Feature screening was initially [13] introduced for a linear model through marginal independence learning based on the Pearson correlation. The screening mechanism asymptotically almost surely identifies all important predictors, and thus is called “sure independence screening” (SIS). Since in many applications researchers know from previous investigations that certain features are responsible for the outcomes or should be controlled for in the studies, conditional SIS [14] (CSIS) that allows multiple covariates to be adjusted for was also proposed for linear models. Although SIS and CSIS have been substantially extended to handle different types of data including right-censored survival outcomes [15], not much attention has been attracted to the competing risks data.

In this paper, we propose a conditional gene screening procedure for the PSH model, controlling for important clinical covariates. The procedure can be combined with available stepwise selection [16], penalized or boosting methods to identify a short list of influential genes and build an interpretable prognostic model for subsequent inference. Although the goal of this paper is variable screening, predictive models were built to evaluate the selected variables, and to compare our proposal with existing selection methods. The screening step eases the computational burden of the penalized or boosting approaches, leading to an enhancement of their performance. We were particularly interested in applying the proposed procedure to discover a predictive gene signature for progression in early-stage bladder cancer. We performed screening followed by boosting and validated the prognostic value of the resulting gene signature after adjusting for the effect of some clinical covariates. As will be shown, this screening-and-selection paradigm has computational and statistical advantages over classical selection tools for high-throughput CR data. The proposed procedure can be easily implemented with available R packages for CR regression, and readily applied to other cancer datasets.

## 2. Materials and Methods

### 2.1. The Proportional Subdistribution Hazards Model

Let *T* and *C* be the failure and censoring times, and ϵ∈{1,…,K} be the cause of failure. Let $X\in R^{p}$ denote the vector of *p* covariates subject to selection and $Z\in R^{p_{0}}$ denote the vector of $p_{0}$ covariates to be controlled for in the analysis. For typical right-censored data, we observe Y=min(T,C) and δ=I(T≤C), where I(·) is the indicator function. Our goal is to model the cumulative incidence function (CIF) for failure from the cause of interest (ϵ=1) conditional on the covariates:

$$F_{1}\left(t;X,Z\right)=Pr\left(T\leq t,\epsilon =1|X,Z\right),$$

i.e., the probability of experiencing event 1 before time *t* and before the occurrence of any other types of event. The subdistribution hazard [17] associated with event 1 is defined as

$$\begin{array}{cc} \lambda_{1}\left(t;X,Z\right) & =\lim_{\Delta t\to 0}\frac{Pr(t\leq T\leq t+\Delta t,\epsilon =1|T\geq t\cup (T\leq t\cap \epsilon \neq 1),X,Z)}{\Delta t}\\ \; & =-\frac{d\log(1-F_{1}\left(t;X,Z\right))}{dt} \end{array}$$

which measures the instantaneous risk of failure from event 1 for patients who have not yet experienced the event. Note that this risk set includes those who are currently event free as well as who have previously experienced a competing event. The subdistribution hazard for event 1 is assumed to follow a proportional hazard model [5]

$$\lambda_{1}\left(t;X,Z\right)=\lambda_{1,0}\left(t\right)\exp\left(\beta^{T}X+\gamma^{T}Z\right),$$

where $\lambda_{1,0}\left(t\right)$ is an unspecified baseline hazard, and $\beta \in R^{p}$ and $\gamma \in R^{p_{0}}$ are regression coefficients. Given a finite sample ${\left\{X_{i},Z_{i},Y_{i},\delta_{i},\epsilon_{i}\right\}}_{i=1}^{n}$, the coefficients can be estimated by maximizing the log partial likelihood function

$$l_{n}\left(\beta ,\gamma \right)=\sum_{i=1}^{n}\int_{0}^{\infty }\left\{\beta^{T}x_{i}+\gamma^{T}z_{i}-\log\sum_{j=1}^{n}w_{j}\left(t\right)R_{j}\left(t\right)\exp\left(\beta^{T}x_{j}+\gamma^{T}z_{j}\right)\right\}w_{i}\left(t\right)dN_{i}\left(t\right),$$

where $N_{i}\left(t\right)=I\left(Y_{i}\leq t,\epsilon_{i}=1\right)$, $R_{i}\left(t\right)=1-N_{i}\left(t-\right)$, and $w_{i}\left(t\right)=I\left(C_{i}\geq Y_{i}\wedge t\right)\hat{G}\left(t\right)/\hat{G}\left(Y_{i}\wedge t\right)$ are weights to account for censoring with $\hat{G}\left(t\right)$ being the Kaplan–Meier estimate for the censoring time G(t)=Pr(C≥t). Denote the maximizer by $\left(\hat{\beta },\hat{\gamma }\right)=\arg\max_{\beta ,\gamma }l_{n}\left(\beta ,\gamma \right)$. Having obtained the estimated regression coefficients, the estimated CIF is obtained by

$${\hat{F}}_{1}\left(t\right)=1-\exp\left(-{\hat{H}}_{1,0}\left(t\right)\exp\left({\hat{\beta }}^{T}x_{j}+{\hat{\gamma }}^{T}z_{j}\right)\right),$$

where ${\hat{H}}_{1,0}\left(t\right)$ is the Breslow estimator of the cumulative baseline subdistribution hazard. The prediction error of the estimated CIF can then be calculated as

$$Err\left(t\right)=\frac{1}{n}\sum_{i=1}^{n}{\left(N_{i}\left(t\right)-{\hat{F}}_{1}\left(t\right)\right)}^{2}W_{i}\left(t\right)$$

after accounting for censoring, which is often used to evaluate the performance of the fitted PSH model. Here, $W_{i}\left(t\right)=\frac{I\left(Y_{i}\leq t\right)I\left(Y_{i}\leq C\right)}{\hat{G}\left(Y_{i}-|X_{i}\right)}+\frac{I(Y_{i}>t)}{\hat{G}\left(t|X_{i}\right)}$ is the inverse probability of censoring weights. The estimation, prediction and evaluation of the PSH model can be achieved via the R packages riskRegression and pec. However, the partial likelihood estimation is no longer applicable when $p+p_{0}>n$, demanding new techniques to be developed for high dimensional settings.

### 2.2. Conditional Sure Independence Screening for PSH

For high dimensional data, we realistically assume that the true parameter $\beta =(\beta_{1},\ldots ,\beta_{p})$ is sparse. In other words, the subset

$$X_{A}=\left\{X_{j}:\beta_{j}\neq 0,j=1,\ldots ,p\right\}$$

is small. Our aim is therefore to identify the active subset $X_{A}$ and estimate β. Approaches have been developed to obtain a sparse model by maximizing a penalized likelihood function [6,7,8,9,10]. Component-wise boosting [11] is an alternative way to obtain parsimonious model fits, and has been adapted to the PSH setting [12]. It uses a stepwise procedure that allows us to build up an overall model from many simple fits, and in each boosting step, the coefficient for one predictor is updated and the overall fit is refined. Boosting and LASSO-like penalized methods are known to have deep connections [11], and they produce similar sparse solutions. However, the aforementioned methods may become statistically inaccurate and computationally expensive when $p+p_{0}>>n$ [13]. In the next section, we introduce a feature screening procedure for the PSH model to address the issue.

**Figure 1:**
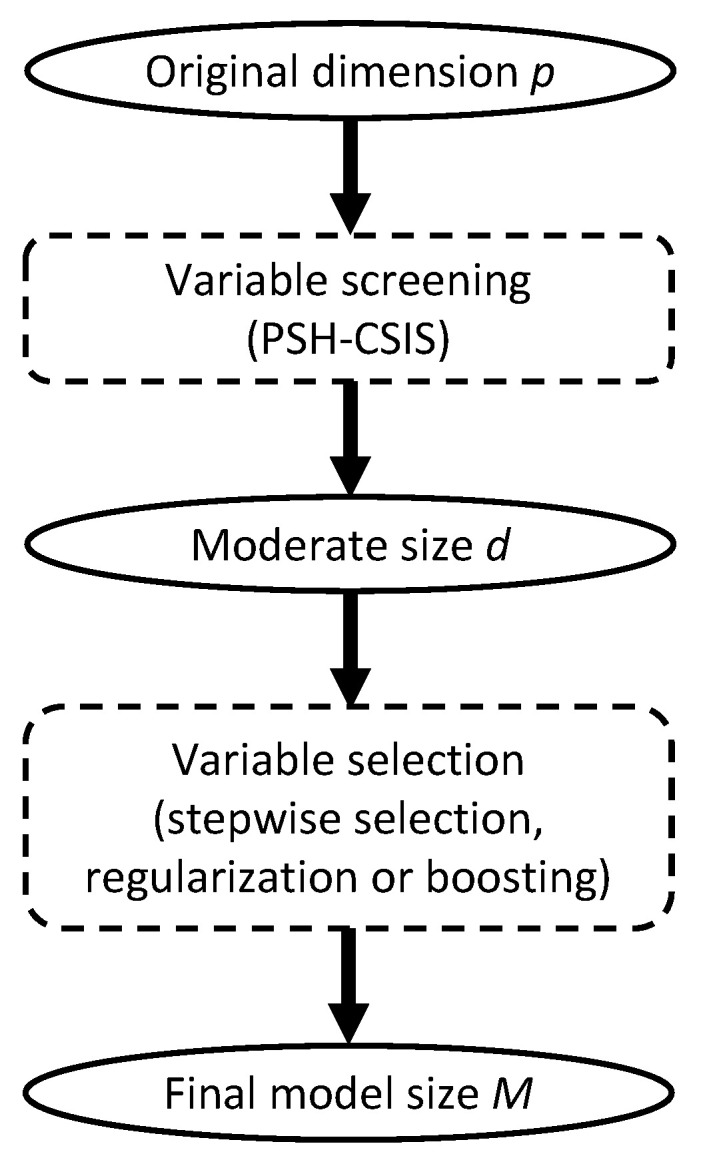
Overall diagram of variable screening and selection.

For each j=1,…,p, consider the PSH model containing an individual predictor $X_{j}$ in addition to the control variables

$$\lambda_{1}\left(t;X_{j},Z\right)=\lambda_{1,0}\left(t\right)\exp\left(\beta_{j}X_{j}+\gamma^{T}Z\right),$$

and let

$${\hat{u}}_{j}=\max_{\beta_{j},\gamma }l_{n}\left(\beta_{j},\gamma \right)$$

denote its estimated maximum log partial likelihood. Then, ${\hat{u}}_{j}$ can be regarded as the change in likelihood associated with $X_{j}$ given Z, after ignoring the common constant $\max_{\gamma }l_{n}\left(\gamma \right)$. That is, ${\hat{u}}_{j}$ measures the marginal contribution of $X_{j}$ to the survival outcome after adjusting for the effect of Z, and a large value suggests $\beta_{j}\neq 0$. We therefore propose to recruit the variables

$${\hat{X}}_{A}=\left\{X_{j}:{\hat{u}}_{j}\;\mathrm{is}\;\mathrm{among}\;\mathrm{the}\;\mathrm{first}\;d\;\mathrm{largest}\;\mathrm{of}\;\mathrm{all}\right\}$$

for a pre-specified model size *d*. We henceforth refer to the above procedure as conditional sure independence screening for PSH model, or PSH-CSIS for short. According to the sure screening property [13], the choice of *d* can be relatively generous to ensure that all the important predictors are preserved with high probability. Conventional choices of *d* are [n/log(n)], 2[n/log(n)], 3[n/log(n)], and $n-p_{0}-1$ [13,18]. Once the dataset is sufficiently downsized by PSH-CSIS, existing lower dimensional methods can be used afterwards for more precise variable selection and statistical inference (Figure 1).

### 2.3. Non-Muscle-Invasive Bladder Carcinoma Data

Gene expression data and clinical data for patients with NMI bladder carcinoma were acquired from GEO database (accession number GSE5479 [19]). In total, 300 patients with complete information on 1381 microarray features and 5 important clinical covariates (age, sex, reevaluated WHO grade, reevaluated pathological disease stage and BCG/MMC treatment) were included for analysis. Table 1 summarizes the 5 clinical covariates. The primary endpoint, the time to progression or death from bladder cancer, was observed for 83 patients. Besides, 33 patients died from other/unknown causes and 184 patients were censored during follow-up. The progression-free survival time ranges from 0 to 185 months, with a median of 47 months.

The patients were divided into training and testing subgroups (4:1 ratio), such that the two cohorts share similar clinical characteristics. The sampling procedure was conducted via the R package SPlit. The training cohort was comprised of 240 samples and the testing cohort was comprised of 60 samples. We first performed PSH-CSIS on the training subset and pre-selected 240−1−5=234 genes after adjusting for the effect of the clinical covariates. The likelihood-based boosting approach [12] (CoxBoost) was then applied to the reduced training data for further gene selection and prognostic modeling simultaneously through the R package CoxBoost. The clinical covariates remained unpenalized in the boosting procedure and the optimal tuning parameters were determined through 10-fold cross validation. The training and testing prediction errors of the estimated CIF were calculated. As the apparent error evaluated on the training data will underestimate the true prediction error, bootstrap .632+ prediction error [12] was calculated instead. Besides, the time-dependent receiver operating characteristic (ROC) curve along with the area under the curve (AUC) were obtained on the testing data using the R package riskRegression. A patient’s risk score was defined as the linear combination of the selected genes where the coefficients were extracted from the fitted model (i.e., the gene signature values). The risk score was used to classify the patient as having high or low risk, with the median score of the training group being the cutoff. The same cutoff value was also applied when assigning the test samples. The two risk groups were contrasted by cumulative incidence analysis [17] via the R package cmprsk. The performance of the proposed model is compared with a direct boosting procedure without screening as well as a PSH model containing only the clinical covariates. All three models were benchmarked against a null model of the Aalen–Johansen estimator that employs no genetic or clinical covariates. Finally, a PSH model was fitted to the entire dataset to conduct inference about independent prognostic factors associated with progression, where the gene signature identified by the PSH-CSIS+CoxBoost model and the five clinical covariates were used. As pointed out by a reviewer, age ≥ 70 is a potential risk factor for NMIBC. Hence, age was dichotomized at 70 in the final model. As suggested by another reviewer, two additional models were considered for sensitivity analysis: (a) PSH with Lasso penalty and (b) PSH-CSIS followed by PSH with Lasso penalty, using the same analysis scheme described above. The R package fastcmprsk was used for fitting the Lasso model. R code for implementing the PSH-CSIS+CoxBoost model is available at https://github.com/cke23/GeneScreeningBLCA (accessed on 10 October 2022).

## 3. Results

Table 2 lists the influential genes selected by the PSH-CSIS + CoxBoost model and the only CoxBoost model. The two models selected five genes in common and post-screening CoxBoost identified an additional influential gene. Computing times for running the PSH-CSIS + CoxBoost model and the CoxBoost model (including parameter tuning via cross validation and model fitting) were 2.95 min and 4.04 min, respectively, on a laptop with an i5 1.4 GHz processor and 16 G RAM, which suggests the computational benefit of performing screening before boosting.

**Figure 2:**
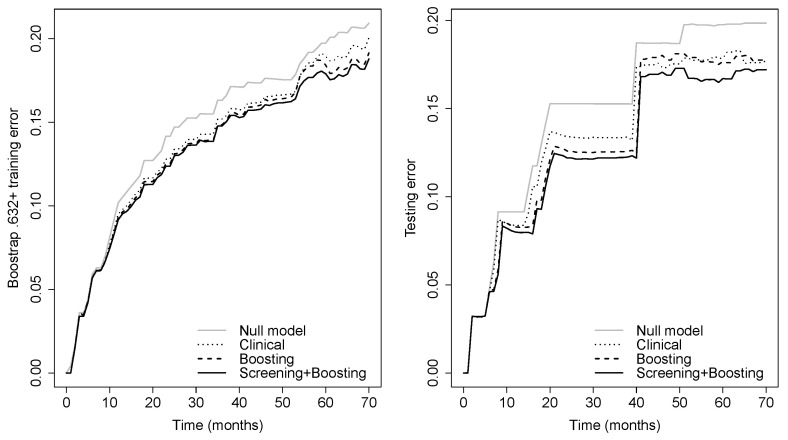
Bootstrap .632+ training error curve (**left panel**) and testing error curve (**right panel**) for prediction of the cumulative incidence function.

**Figure 3:**
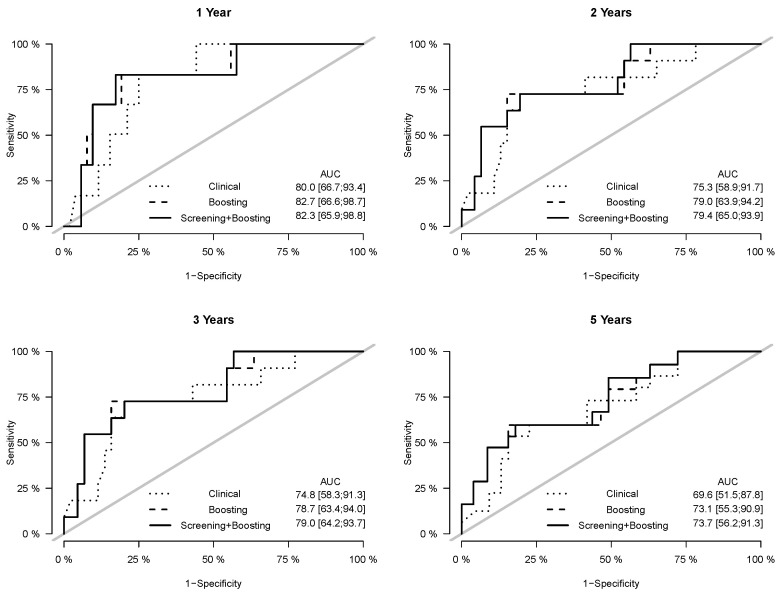
ROC curves and assocaited AUC values of the cumulative incidence predicted by the competing models on the testing data, at years 1, 2, 3 and 5.

**Figure 4:**
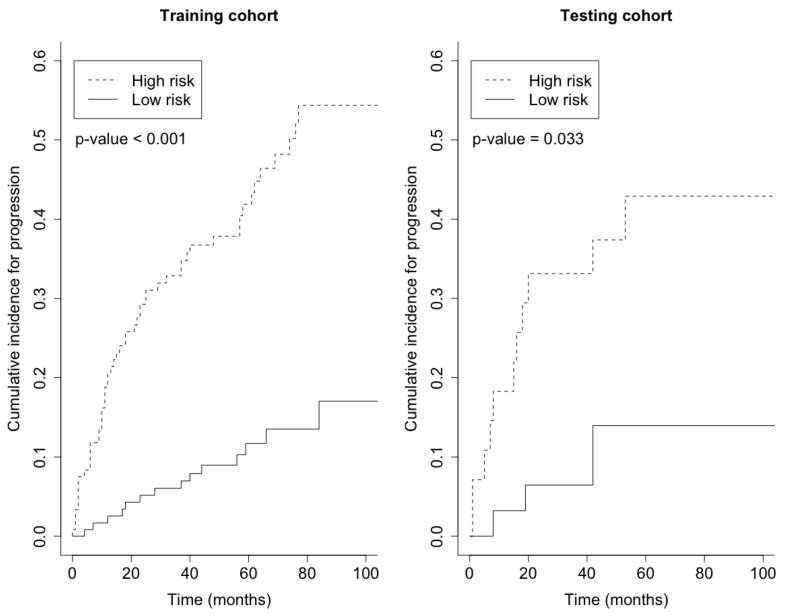
Cumulative incidence by risk group on the basis of the gene signature identified by the PSH-CSIS+CoxBoost model.

Prediction error curves of the CIF for all models are displayed in Figure 2. The purely clinical model (dot curves) is seen to clearly improve over the null model (grey curves), indicating that valuable information is contained in the clinical covariates. Incorporating genetic information from the microarray data further assisted in predicting progression, as shown by the CoxBoost model (dash curves). The PSH-CSIS+CoxBoost model (solid curves) performed the best among all models. A similar observation can be found on the ROC curves of the predicted cumulative incidence at 1, 2, 3 and 5 years on the testing data, which are displayed in Figure 3. In addition, cumulative incidence analyses revealed that the gene signature identified by the PSH-CSIS+CoxBoost model provides effective risk stratification (*p*-value < 0.001 for the training cohort and *p*-value = 0.033 for the testing cohort; Figure 4).

From the multivariable PSH model fitted to the whole data (Table 3), the six-gene signature selected by PSH-CSIS+CoxBoost was a significant predictor with an hazard ratio of 12.55 (*p*-value < 0.001), adjusted for other clinical covariates. The hazard of progression on average increased by 55% after age 70 (*p*-value = 0.058). Having a low grade tumor led to a reduction of hazard by 57% (*p*-value = 0.004) compared to high grade tumors (*p*-value = 0.005). Diagnosis at an early stage decreased the hazard of progression by 40% (*p*-value = 0.056). Receiving BCG/MMC treatment also resulted in a reduction of hazard by 62% (*p*-value = 0.002). The high hazard ratio associated with the six-gene signature reflects the strong genetic effect on survival from progression given the clinical covariates already in the model. The effectiveness of the conditional screening-and-selection procedure is thus demonstrated.

**Figure 5:**
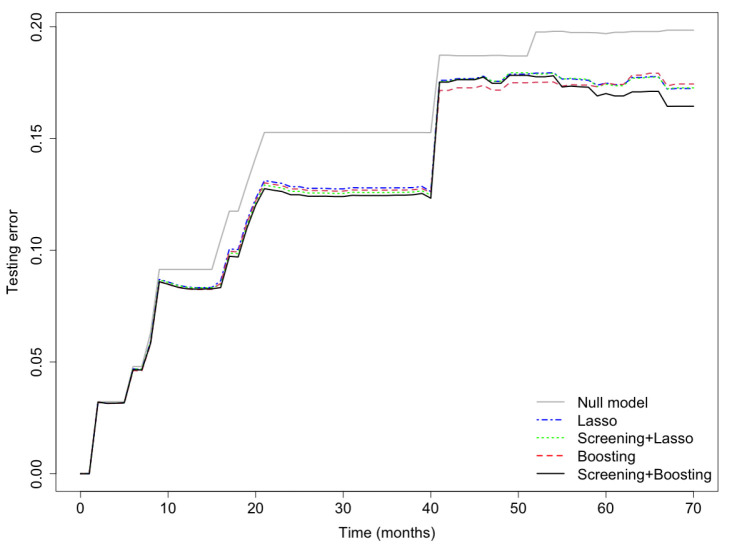
Sensitivity Analysis: testing error curves for the cumulative incidence predicted by the competing models.

**Figure 6:**
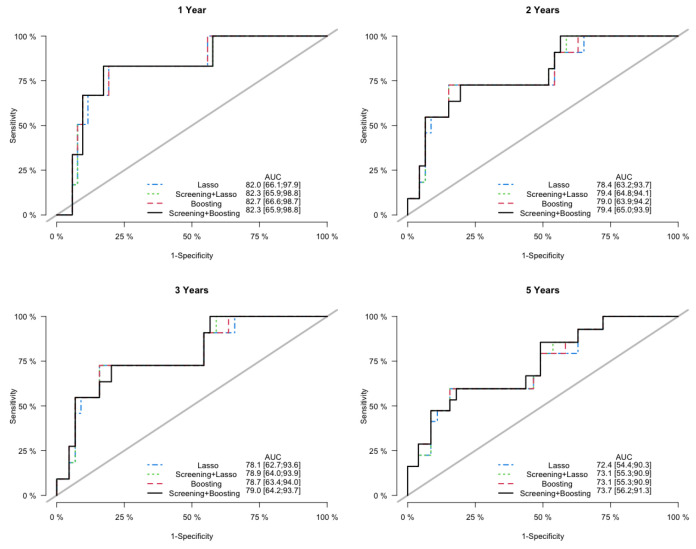
Sensitivity Analysis: ROC curves and assocaited AUC values of the cumulative incidence predicted by the competing models on the testing data, at years 1, 2, 3 and 5.

Finally, in the sensitivity analysis, the Lasso model and the PSH-CSIS+Lasso model selected the same 5 genes (AP1M2, CAT, CCL3, MCM7, NCF2) as the CoxBoost model. Furthermore, their predictive performances were also similar to the CoxBoost model, but inferior to the PSH-CSIS+CoxBoost model. Prediction error curves of the cumulative incidence for the competing models are displayed in Figure 5. In addition, Figure 6 displays the ROC curves of the predicted cumulative incidences at 1, 2, 3 and 5 years on the testing data. We observe that the PSH-CSIS+Lasso model is better than the Lasso-only model in terms of AUC for all the years, while the PSH-CSIS+CoxBoost model is superior to the PSH-CSIS+Lasso model for the 3-year and 5-year predictions. Computing times for running the Lasso model and the PSH-CSIS+Lasso model (including parameter tuning via cross validation and model fitting) were 11.32 min and 2.82 min, respectively, on a laptop with i5 1.4 GHz processor and 16 G RAM. Although the two models selected identical genes and made similar predictions, the computational gain using the latter is substantial.

## 4. Discussion

We first highlight some biological insights associated with the genes selected by PSH-CSIS+CoxBoost. Adaptor related protein complex 1 subunit mu 2 (AP1M2) is a component of clathrin adaptor complex, which is required for maintaining correct polarity of basolateral membrane proteins in epithelial cells [20]. As yet, there are no functional studies interrogating the role of AP1M2 in cancer. Bioinformatic analyses on AP1M2 are not conclusive, e.g., its level has no effect on patient survival in pancreatic cancer [21], it was identified as a hub gene in renal cancer in which its expression is downregulated [22], while its high expression predicted poor outcome in invasive breast carcinoma [23]. AP1M2 is required for maintaining cell polarity, a marker of differentiation. Dedifferentiation is a hallmark of cancer and it might be anticipated that loss of AP1M2 will prevent proper polarization of cells, thereby contributing to dedifferentiation. The observation that low levels of AP1M2 is associated with poor prognosis in bladder cancer patients is, therefore, meaningful. Reactive oxygen species (ROS) create oxidative stress, resulting in mutagenesis and tumor pathogenesis. The antioxidant enzyme catalase (CAT) provides protection against oxidative stress and downregulation or inactivity of catalase is observed in many cancers [24]. A decreased catalase expression in cancerous bladder tissues in comparison with normal tissues and its association with disease recurrence have been reported by several studies [25,26]. Plakophilin 4 (PKP4), also known as p0071, belongs to the family of armadillo-like proteins, and is involved in regulating adherens junction organization [27]. Overexpression of PKP4 in A431 cells inhibited the ability to close in vitro wounds, suggesting decreased motility of the cells [28]. As yet, the role of PKP4 in bladder cancer is not known and other members of this family play a variable role, e.g., PKP2 is upregulated and PKP3 is downregulated in invasive bladder cancer [29]. It is possible that low levels of PKP4 facilitate increased the motility of bladder cancer cells, conferring an aggressive, metastatic disease. Chronic inflammation plays a key role in bladder carcinogenesis [30]. The cytokine C-C motif chemokine ligand 3 (CCL3), also known as macrophage inflammatory protein 1 alpha (MIP1A), functions in the tumor microenvironment by recruiting tumor-associated macrophages (TAMs) and myeloid-derived suppressor cells (MDSC), thus creating an immunosuppressive environment and facilitating metastasis [31]. CCL3 has been shown to be produced by myeloid cells in bladder cancer patients, which contribute to the pathogenesis of inflammation and immunosuppression [32]. Mini-chromosome maintenance proteins (MCM) are key components of pre-replication complex and are essential for genome replication thereby functioning as oncogenes [33]. A member of this family, MCM7, has been shown to be up-regulated in numerous cancers including bladder cancer [34,35], especially in human papillomavirus (HPV)-positive tumors [36]. Neutrophil cytosolic factor 2 (NCF2) is a subunit of NADPH complex found in neutrophils. NCF2 plays a role in inflammation and cancer, e.g., it is highly expressed in gastric cancer promoting tumor metastasis and invasion by activating NF-κB signaling [37]. In bladder cancer, a high expression level of NCF2 was shown to be associated with an undesirable abundance of chemokine CXCL8, a marker of immunosuppressive MDSCs [38]. Overall, it might be inferred that the expression pattern of our six gene signature predicts a poor prognosis based on what is known about the function of each gene. Nonetheless, further studies, such as multi-center validation, are needed to quantify a stronger evidence and make these new discoveries feasible for use in clinical practice.

In addition to progression, recurrence (in ≤80% of patients) is another important issue in NMIBC, especially for patients with non-invasive papillary carcinoma (Ta). Several clinical and pathological factors of recurrence have been identified such as multiplicity, tumor size, and prior recurrence rate [39]. A variety of markers has also been linked to recurrence in bladder cancer, although the role for molecular markers to predict recurrence seems limited because multifocal disease and incomplete treatment are probably more important for recurrence than the molecular features of a removed tumor [40]. Recurrence information is not available in the dataset that we studied in this paper. However, we expect that similar analysis can be carried out to discover molecular drivers, if the survival outcome for recurrence is provided. Metabolomic studies in bladder cancer for diagnosis and prognosis are also characterized by high dimensional data. Based on different available samples (urine, blood, tissue sample), advances in molecular pathology have driven efforts to identify prognostic and predictive markers and classify bladder cancer into subtypes [41]. Potential applications of the proposed screening procedure in metabolomic data analysis for diagnostic management and pathologic profiling are worthy of future research.

The proposed screening procedure is generalizable, can be combined with other existing feature selection techniques for the PSH model, and applied to other types of cancer data containing competing events. The method can also be adapted to some variations of the PSH model. In practice, the assumption of proportional hazards is unlikely to be satisfied for all covariates in a high-dimensional setting. Bellach et al. [42] thus developed a weighted likelihood function that generalizes the PSH model by allowing time-dependent covariate effects on the subdistribution hazard. On the other hand, control variables such as age can have dynamic impacts on the survival time, motivating us to consider a varying coefficient model to assess nonlinear interaction effects between the primary covariates and control variables [43]. Screening procedures to be developed for these more flexible models may lead to improved model fit and new marker discoveries.

## 5. Conclusions

The development of high-throughput biology provides a vast amount of information about various phenotypic data, including survival endpoints. Finding prognostic gene signatures for cancer survival became a vital task in biomedical research. The challenge is often threefold. First, the number of features in microarray or sequencing data is much larger than the sample size, creating a predicament to the use of classical statistical analysis tools. How to eliminate noise variables while preserving important features is the key to successful downstream analysis. Although penalized methods have been widely used for feature selection, they can be unstable and computational expensive for high-throughput data. Fast and effective feature screening tools for very high dimensional data have been emerging in the past decade in the statistics literature. However, the dissemination of these attractive methods to biomedical fields is limited. Second, survival endpoints are complicated by censoring and events other than that of primary interest. Data analysis including feature screening/selection must be tailored for the specific survival setting. Third, the desire of incorporating both clinical and genetic information also adds to the difficulty of prognostic modeling. One also has to tradeoff between prediction accuracy and model interpretability.

In this paper, we aimed to address the aforementioned issues for high-throughput competing risks data, in the context of bladder cancer prognosis. The PSH model proposed by Fine and Gray is commonly used for studying survival among competing events, and its estimation and inference has been well-studied. We therefore proposed a feature screening procedure for the PSH model and suggested an analysis pipeline of screening-and-selection for identifying influential genes while controlling for important clinical covariates. The implementation is straightforward with existing R packages for competing risks regression. It was demonstrated that our approach was able to reduce the dimension efficiently and improve the performance of the subsequent boosting procedure for feature selection. In particular, we identified a predictive six-gene signature, independent of numerous clinical covariates, for progression in early-stage bladder cancer. The gene signature was shown to be numerically effective and biologically sensible, but multi-center validation is needed to better evaluate its potential value in clinical practice.

## Figures and Tables

**Table 1:** Summary of clinical and pathological characteristics.

Variables	Frequency (Percent)
Age	
Less than 60	42 (14.0%)
60–69	67 (24.0%)
70–79	105 (35.0%)
80 or greater	81 (27.0%)
Gender	
Female	59 (19.7%)
Male	241 (80.3%)
WHO Grade	
High	176 (58.7%)
Low	124 (41.3%)
Stage	
Ta	173 (57.7%)
T1	127 (42.3%)
Treatment	
BCG/MMC	82 (27.3%)
None	218 (72.7%)

**Table 2:** Genes selected by the two competing models. A risk gene with a positive coefficient from the fitted model is denoted by “+”, while a protective gene with a negative coefficient is denoted by “-”.

Model	Gene Selected
PSH-CSIS + CoxBoost	AP1M2(-), CAT(-), CCL3(+), MCM7(+), NCF2(+), PKP4(-)
CoxBoost	AP1M2(-), CAT(-), CCL3(+), MCM7(+), NCF2(+)

**Table 3:** Hazard ratio estimates, 95% confidence intervals (CI) and *p*-values for the 6-gene signature (identified by PSH-CSIS+CoxBoost) and other clinical covariates, corresponding to cumulative incidence function of Cause 1 (progression, or death from bladder cancer), obtained from fitting the univariate or multivariable competing risks Cox regression.

Variable	Univariate Analysis		Multivariable Analysis	
	Hazard Ratio (95% CI)	*p*-Value	Hazard Ratio (95% CI)	*p*-Value
6-gene signature	8.95 (4.75, 16.90)	<0.001	12.55 (6.11, 25.80)	<0.001
Age				
Age > 70	1.70 (1.11, 2.60)	0.015	1.55 (0.99, 2.45)	0.058
Age ≤ 70	-	-	-	-
Gender				
Male	0.80 (0.43, 1.33)	0.38	0.84 (0.46, 1.55)	0.580
Female	-	-	-	-
WHO Grade				
low	0.40 (0.28, 0.64)	<0.001	0.43 (0.24, 0.77)	0.005
high	-	-	-	-
Stage				
Ta	0.63 (0.41, 0.97)	0.034	1.66 (0.99, 2.80)	0.056
T2	-	-	-	-
Treatment				
None	2.04 (1.19, 3.48)	0.009	2.60 (1.44, 4.69)	0.002
BCG/MMC	-	-	-	-

## Data Availability

https://www.ncbi.nlm.nih.gov/geo/query/acc.cgi?acc=GSE5479 (accessed on 30 August 2022).
